# Avoidant Restrictive Food Intake Disorder (ARFID) and Body Image: a case report

**DOI:** 10.1186/s40337-022-00583-0

**Published:** 2022-05-04

**Authors:** Angela Barney, Lindsey D. Bruett, Sarah Forsberg, Jason M. Nagata

**Affiliations:** 1 Division of Adolescent and Young Adult Medicine, Department of Pediatrics, University of California San Francisco, 3333 California Street, Suite 245, San Francisco, CA 94118 USA; 2 Eating Disorders Program, Department of Psychiatry, University of California San Francisco, 401 Parnassus Ave, San Francisco, CA 94143 USA

**Keywords:** Avoidant Restrictive Food Intake Disorder, Body image, Body dissatisfaction, Adolescent, Case report

## Abstract

**Background:**

Avoidant Restrictive Food Intake Disorder (ARFID) is a relatively new eating disorder diagnosis, and there is need to better understand this disorder’s presentation. Diagnostic criteria for ARFID require that there are no body image distortions. People with ARFID symptoms may have body image concerns that require careful consideration and more information about the interplay of these is needed to help clinicians appropriately diagnose and manage ARFID.

**Case presentation:**

This clinical observation reports a case of ARFID in a nine-year-old with severe malnutrition who positively views her small size and values thinness. The patient reported that her own desire for thinness was influenced by social media beauty ideals and praise of thinness witnessed in social situations. Despite this, the motivation for avoidant and restrictive eating behaviors was low appetitive drive, fear of trying new foods, and fear of adverse consequences from eating.

**Conclusions:**

Although concerning, the patient’s body image was not of clinical significance as a motivating factor for the disordered eating behaviors. Body image dissatisfaction is common. The requirement to exclude body image distortions in the diagnostic criteria for ARFID may require consideration of the pervasiveness of societal body ideals to which young people are exposed.

## Background

Avoidant Restrictive Food Intake Disorder (ARFID) is a new diagnosis first included in the Diagnostic and Statistical Manual of Mental Disorders, 5th edition (DSM-5) [1]. ARFID is characterized by disordered eating behaviors without body image disturbance. While variants of ARFID presentations have been described in the literature, evidence regarding unique challenges in the diagnosis and management of this disorder remains limited [2]. There is a need to further understand its heterogeneous presentation in order to inform future work towards development of treatment strategies [3].

This paper highlights a case of a 9-year-old girl with malnutrition and chronic symptoms consistent with ARFID in addition to expressed positive attitude toward her small size and desire to maintain thinness. Although diagnostic criteria for ARFID require “no evidence of disturbance in the way in which one’s body weight or shape is experienced,” given the high community prevalence of weight and shape concerns among children and adolescents [4] this requirement may confuse the diagnosis of ARFID. Although specialized clinicians who frequently diagnose ARFID may be well-versed in the nuances of assessing the significance of body dissatisfaction, the underlying motivation of disordered eating may still be difficult to assess for practitioners less familiar with this diagnosis. The patient and her parent gave permission to publish the following case report.

## Case report

The patient is a nine-year-old female who was admitted to a pediatric hospital medicine service with malnutrition (body mass index Z-score of -2.14), stunted growth (height Z-score of -2.28), and several medical complications of malnutrition including xerophthalmia secondary to vitamin A deficiency, vitamin B12 deficiency, and iron deficiency anemia. The cause of malnutrition was thought to be inadequate food intake alone after other organic causes were ruled out. Organic work-up included endoscopy to rule out signs of inflammatory bowel disease or eosinophilic esophagitis, thyroid function studies, sweat chloride test, pancreatic elastase, and celiac panel.

The patient displayed the three characteristics of ARFID presentation to varying degrees. Her mother first noted significant picky eating at age two, which further worsened over time. By early school-age years she would exclusively eat specific foods, limited to popcorn, mashed potatoes, French fries, potato chips, and ramen soup. For each of these there was only one or two brands of the foods that she was willing to eat. The patient became markedly distressed when presented with a new food to try, stating “I’m afraid I won’t like it and I’ll spit it out.” She displayed a low appetitive drive and stated that often she just is not interested in food. Her mother described the patient requiring reminders to eat including need for teachers at school to supervise her lunch and encourage her to eat. She reported that she sometimes is unable to try new foods due to specific smells, tastes or textures. At times, the patient endorsed a fear of vomiting or having an allergic reaction to foods, although this seemed to be inconsistent. The patient and her mother both described these symptoms to be impairing to daily functioning, including limiting social opportunities with peers, limiting patient’s participation in certain family outings due to food selection available.

In addition, the patient’s mother noted rare but consistent comments by the patient regarding body image. For example, when watching a football game on television, she exclaimed that she wanted to look like the cheerleaders. When interviewed about body size and weight, the patient endorsed that she liked being small, noting positive comments she has received from both peers and adults. She also cited YouTube as a source of her positive attitude toward thinness. She and her mother denied body avoidance or checking, or any compensatory behaviors aimed at weight or shape such as compulsive exercise. Further, it consistently appeared (through observation during hospitalization and per mother report) that sensory concerns, low appetitive drive, and at times, fear of vomiting/allergic reaction were the source of eating challenges, rather than restriction aimed at weight loss.

She was assessed using the parent version of the Eating Disturbances in Youth Questionnaire (P-EDY-Q) and the Parent Eating Disorder Examination Questionnaire (PEDE Q) [5, 6]. The P-EDY-Q (by maternal responses) was consistent with ARFID for avoidance of foods due to appearance/texture/smell, lack of interest in food, low weight, and for one component of the rule-out of distorted cognition. For the other distorted cognition component, her score was three for item 20, “My child feels fat, even if I do not agree.” Less than three is considered consistent with ARFID). The PEDE Q was reassuring against significant concerns about weight or shape (global score of 0.519). She scored zero for dissatisfaction with her weight (item 25) or shape (item 26), weight (item 22) or shape (item 23) influencing how she thinks about herself as a person, or weight or shape making it difficult to concentrate on things she is interested in (item 8). Given these findings and the lack of evidence that body image concerns were motivating her limited nutrition intake, she was ultimately diagnosed with ARFID.

Following discharge, the patient was treated as an outpatient by an interdisciplinary team specializing in eating disorders. During this period, the patient demonstrated a positive response to weight gain. She found praise reinforcing and at times expressed excitement when gaining weight.

## Discussion and conclusions

This case illustrates an example of malnutrition resulting from ARFID, despite some body image concerns. In arriving at an appropriate diagnosis for the patient, clinicians considered longstanding and clinically significant concerns in all three domains of ARFID [1]. Though the patient’s body image concerns warranted careful monitoring, they did not appear to be influencing the patient’s behaviors. Therefore, while worrisome, these concerns were conceptualized as within normal limits for this patient within her environmental context. Specifically, she expressed a positive attitude toward her small size and reported influence from social media on her outlook regarding thinness. This case illustrates that ARFID may co-occur with an over-valuing of thinness influenced by media and societal body ideals, but that, ultimately, the apparent underlying motivation for the disordered behaviors and the developmental history of eating concerns must be carefully assessed.

Social media may be associated with body image dissatisfaction and disordered eating behaviors [2, 7]. One study of school-aged children showed that viewing photos of celebrities may be associated with similar body dissatisfaction previously described in adolescent girls [8]. Body image dissatisfaction, even among school-age children, is common. Studies have found that roughly half of girls age 9–12 would like to be thinner [4]. Nonclinical community samples of adolescent girls have demonstrated evidence of shape and weight concerns [9]. Given this, the exclusion of disturbance in weight or shape experience that is required for the diagnosis of ARFID should be considered in the context of the pervasiveness of desired thinness in current Western culture and that this may be present in school-age children, in addition to ARFID. Clinicians should consider the high prevalence of body dissatisfaction in the community when assessing whether these concerns should preclude an ARFID diagnosis.

Furthermore, this case suggests that small body size secondary to ARFID may be reinforced by societal feedback or incorporated into one’s self-identity. Although these issues should be addressed and patients should be monitored for development of symptoms of other eating disorders like anorexia nervosa, they need not limit treatment of the primary problem of ARFID. More research is needed to understand the risk for development of other disordered eating behaviors among patients who are diagnosed with ARFID.

## Data Availability

Not applicable.

## Abbreviations

ARFID: Avoidant Restrictive Food Intake Disorder
DSM-5: Diagnostic and Statistical Manual of Mental Disorders, 5th edition
EDY-Q: Eating Disturbances in Youth Questionnaire
PEDE Q: Parent Eating Disorder Examination Questionnaire
